# The Annual Burden of Seasonal Influenza in the US Veterans Affairs Population

**DOI:** 10.1371/journal.pone.0169344

**Published:** 2017-01-03

**Authors:** Yinong Young-Xu, Robertus van Aalst, Ellyn Russo, Jason K. H. Lee, Ayman Chit

**Affiliations:** 1 Clinical Epidemiology Program, Veterans Affairs Medical Center, White River Junction, Vermont, United States of America; 2 Department of Psychiatry, Geisel School of Medicine at Dartmouth, Hanover, New Hampshire, United States of America; 3 Health Outcomes and Economics, Sanofi Pasteur, Swiftwater, Pennsylvania, United States of America; 4 Leslie Dan Faculty of Pharmacy, University of Toronto, Toronto, Ontario, Canada; Universidad Nacional de la Plata, ARGENTINA

## Abstract

Seasonal influenza epidemics have a substantial public health and economic burden in the United States (US). On average, over 200,000 people are hospitalized and an estimated 23,000 people die from respiratory and circulatory complications associated with seasonal influenza virus infections each year. Annual direct medical costs and indirect productivity costs across the US have been found to average respectively at $10.4 billion and $16.3 billion. The objective of this study was to estimate the economic impact of severe influenza-induced illness on the US Veterans Affairs population. The five-year study period included 2010 through 2014. Influenza-attributed outcomes were estimated with a statistical regression model using observed emergency department (ED) visits, hospitalizations, and deaths from the Veterans Health Administration of the Department of Veterans Affairs (VA) electronic medical records and respiratory viral surveillance data from the Centers for Disease Control and Prevention (CDC). Data from VA’s Managerial Cost Accounting system were used to estimate the costs of the emergency department and hospital visits. Data from the Bureau of Labor Statistics were used to estimate the costs of lost productivity; data on age at death, life expectancy and economic valuations for a statistical life year were used to estimate the costs of a premature death. An estimated 10,674 (95% CI 8,661–12,687) VA ED visits, 2,538 (95% CI 2,112–2,964) VA hospitalizations, 5,522 (95% CI 4,834–6,210) all-cause deaths, and 3,793 (95% CI 3,375–4,211) underlying respiratory or circulatory deaths (inside and outside VA) among adult Veterans were attributable to influenza each year from 2010 through 2014. The annual value of lost productivity amounted to $27 (95% CI $24–31) million and the annual costs for ED visits were $6.2 (95% CI $5.1–7.4) million. Ninety-six percent of VA hospitalizations resulted in either death or a discharge to home, with annual costs totaling $36 (95% CI $30–43) million. The remaining 4% of hospitalizations were followed by extended care at rehabilitation and skilled nursing facilities with annual costs totaling $5.5 (95% CI $4.4–6.8) million. The annual monetary value of quality-adjusted life years (QALYs) lost amounted to $1.1 (95% CI $1.0–1.2) billion. In total, the estimated annual economic burden was $1.2 (95% CI $1.0–1.3) billion, indicating the substantial burden of seasonal influenza epidemics on the US Veterans Affairs population. Premature death was found to be the largest driver of these costs, followed by hospitalization.

## Introduction

Seasonal influenza epidemics continue to have a substantial public health and economic burden in the United States. On average, it is estimated that over 200,000 people are hospitalized and 23,000 people die from respiratory and circulatory complications associated with seasonal influenza virus infections each year [1–7]. The annual direct medical costs have been found to average $10.4 billion; indirect costs, which include estimated lost earnings due to illness and loss of life, average $16.3 billion [5]. The total economic burden, using projected statistical life values of seasonal influenza epidemics, is estimated to be as high as $87 billion each year.

The burden of illness caused by seasonal influenza varies by age and the presence of preexisting medical conditions. Serious medical complications leading to hospitalizations and deaths are typically greatest among persons aged 65 years and older, as has been shown previously with influenza-attributed hospitalization rates. High-risk persons aged 18 to 49, 50 to 64, and 65 and older illustrated rates of 4.0, 12.3, and 55.6, respectively, per 10,000 person-periods [4]; in contrast, rates among low-risk adults were 0.5, 1.8, and 18.7 per 10,000 person-periods, respectively.

Though the impact of seasonal influenza epidemics has been studied for the general population, there is a paucity of literature describing the burden of illness and associated costs attributed to this viral infection for the US Veterans Affairs population. The Veterans Health Administration of the Department of Veterans Affairs (VA) serves an older patient population that is predominantly male and has a higher disease burden than the general US population [8]. Leveraging previously described methodologies, the objective of this study was to estimate the economic impact of severe influenza-induced illness on US Veterans Affairs population over a five-year period from 2010 to 2014.

## Materials and Methods

### Study data

This study used data from VA, which is the largest integrated healthcare system in the US and provides comprehensive health services to US Veterans. VA has an integrated and unified electronic medical record system (EMR) that contains information about inpatient, outpatient, and emergency department (ED) visits. This includes procedures, surgeries and diagnoses, pharmacy, laboratory results, extended care, vital signs, healthcare-related survey data and mortality for all persons treated within VA. Each patient is assigned a unique identification number that allows longitudinal follow-up.

VA uses a separate electronic Managerial Cost Accounting system (MCA) to capture the cost of healthcare services. Costs are not based on insurance claims, reimbursements or billing data; rather, they are the allocation of actual expenses (e.g. salaries, equipment, buildings, energy, negotiated drug prices, materials) to a specific healthcare encounter. Unlike claims data, such as those used by the Centers for Medicare and Medicaid Services, VA expense data are not subject to variation in coding practices. Data on Veteran mortality are likewise consistent, as VA Vital Status files incorporate information through multiple sources that include the National Death Index (NDI) and capture deaths that occur at VA, as well as non-VA locations, including at home or non-VA facilities.

The study observation period encompassed five respiratory seasons from 2010/11 through 2014/15 (simplified as 2010 to 2014). A respiratory season was defined as starting in calendar week 27 during a given year and ending in calendar week 26 of the following year. For example, respiratory season 2010/11 started June 27, 2010 and lasted through June 25, 2011. The study population was comprised of Veterans aged 18 and older enrolled at VA. To ensure we included those who received non-incidental care, patients were eligible for inclusion in a given respiratory season only if they had at least one inpatient or outpatient VA healthcare encounter during the prior respiratory season. Patients were classified into three groups for each season based on age, which was calculated at the end of the respiratory season or death: 18 to 49, 50 to 64, and 65 and older. Patients were also classified each season as at high-risk or at low-risk for influenza-related complications; this categorization was based on the presence of one or more primary International Classification of Diseases—9th edition (ICD-9-CM) diagnosis codes from an inpatient or outpatient encounter during the prior respiratory season. Patients assigned diagnosis codes for chronic cardiac, pulmonary, renal, metabolic, liver, or neurological diseases; diabetes mellitus; hemoglobinopathies; and/or immunosuppressive conditions and malignancy were at high-risk [4]. Patients for whom we did not find an encounter with one of these diagnosis codes (S1 Table) were classified as at low-risk.

This study was approved by the Veteran’s Institutional Review Board of Northern New England (VINNE) through expedited review for the use of protected health information (PHI) as it was deemed to involve no more than minimal risk to the privacy of individuals (CPHS 28523). All study procedures were carried out in compliance with federal and institutional ethical standards as well as the Helsinki Declaration. The usual requirements for HIPAA authorization and study participants’ written or verbal informed consent were waived by VINNE. Such waivers may be granted by regulatory committees when a research study meets all of the following criteria:

1. The use or disclosure of the PHI involves no more than minimum risk to the privacy of individuals, based on, at least, the presence of all the following elements:
  - An adequate plan to protect the identifiers from improper use and disclosure.
  - An adequate plan to destroy the identifiers at the earliest opportunity consistent with conduct of research, unless there is a health or research justification for retaining the identifiers or such retention is otherwise required by law.
  - Adequate written assurances that the PHI will not be reused or disclosed to any other person or entity, except as required by law, for authorized oversight of the research study, or for other research for which the use or disclosure of PHI would be permitted by the HIPAA Privacy Rule.
2. The research could not practicably be conducted without the waiver or alteration.
3. The research could not practicably be conducted without access to and use of the PHI.

### Estimating influenza-attributed outcomes

Influenza-attributed outcomes were categorized as follows: (1) ED visit; (2) hospitalization; and (3) followed by death (all-cause or with underlying respiratory or circulatory causes). Similar to previously published research methods, a negative-binomial regression model was employed to generate weekly rates for each outcome category by risk and age group as the difference between the model-predicted outcomes and the model-predicted outcomes under the hypothetical absence of influenza (estimated baseline), which were then totaled over the length of the respiratory season [2]. Annual estimates were defined as rates per 100,000 person-periods (respiratory seasons) averaged over the five year study period. The regression model, fit using PROC GENMOD, SAS Enterprise Guide, version 6.1, with the dispersion parameter, was:

$$\begin{array}{l} \text{Y}=\alpha exp\{\beta_{0\;}+\beta_{1}t+{\;\beta }_{2}t^{2}+\beta_{3}t^{3}+\beta_{4}t^{4}+\beta_{5}t^{5}+{\;\beta }_{6}\text{cos}\left(\frac{2\pi t}{52}\right)+{\;\beta }_{7}\text{sin}\left(\frac{2\pi t}{52}\right)\\ +{\;\beta }_{8}\left[\text{A}\left(\text{H}1\text{N}1\right)\right]+\beta_{9}\left[\text{A}\left(\text{H}3\text{N}2\right)\right]+{\;\beta }_{10}\left[\text{A}\left(\text{H}1\text{N}1_{2009}\right)\right]+\beta_{11}\left[\text{B}\right]+\beta_{12}\left[\text{RSV}\right]\} \end{array}$$

where Y represents the weekly number of influenza-attributed outcomes (VA ED visits, VA hospitalizations or all-cause deaths) for each risk and age group, α the offset term (equals to the size of the risk and age group-specific population), β_0_ the intercept, β_1_ the linear time trend, β_2_ through β_5_ the non-linear time trend, β_6_ and β_7_ the seasonal changes in outcomes, β_8_ through β_11_ the weekly percentage of tests positive for each influenza virus type and subtype (*t*) and β_12_ the weekly percentage of tests positive for respiratory syncytial virus (RSV). Publicly available weekly influenza surveillance data, including RSV-specific surveillance data obtained directly from the CDC, were used in the model [9]. Additional parameter details are described in S1 Table.

To estimate excess VA ED visits and VA hospitalizations, only those healthcare encounters at a VA facility with a principal diagnosis of underlying respiratory or circulatory conditions (ICD-9-CM codes 390–519) were included in the model [6–7,10]. The modeled excess deaths initially included those from all causes, and later was adjusted for underlying respiratory and circulatory causes based on a random sample with cause of death.

### Estimating the cost per influenza-attributed encounter

For each influenza-attributed VA ED visit identified, direct medical costs were defined as the sum of the ED costs on the corresponding visit date, including expenses related to office visits, professional consults, prescription medications and laboratory tests, imaging and procedures performed.

VA hospitalizations were categorized by whether or not an episode of extended care followed the discharge. For each influenza-attributed VA hospitalization without extended care identified, direct medical costs were defined as the sum of the inpatient costs from the date of admission to the date of discharge at an acute care facility. For each influenza-attributed VA hospitalization with extended care identified, direct medical costs were defined as the sum of the inpatient, rehabilitation and/or skilled nursing facility costs if the extended care stay began immediately (within 24 hours) following the discharge from an acute care facility.

Direct medical costs for an influenza-attributed encounter defined above were extracted from VA MCA data for those events with a principal diagnosis of underlying influenza and pneumonia (ICD-9-CM codes 480–487) that occurred during each respiratory season [5]. Estimates of encounter-specific direct medical costs were calculated by risk and age group for each respiratory season; results are presented as the average over the five year study period.

### Estimating the economic burden of seasonal influenza epidemics

Data from the US Department of Labor Bureau of Labor Statistics were used to estimate the annual economic burden of productivity loss due to the absence from work [11]. For hospitalizations, the annual estimate of outcome events was multiplied by the 2014 (most resent data available) mean Veteran occupation rates of 73.8%, 64.8%, and 18.8% for age groups 18 to 49, 50 to 64, and 65 and older, respectively; by the 2014 mean daily occupational wage of $182; and by the mean length of stay in days for each risk and age group (S1 Table) [11–12]. For ED visits, a length of stay of one day was assumed. To estimate productivity loss caused by mortality, length of stay was replaced with a three month friction period [13].

To estimate the annual economic burden of influenza-attributed ED visits and hospitalizations, the annual estimate of outcome events was multiplied by the mean cost per outcome event for each risk and age group.

We used previously described methods to estimate the annual economic burden of influenza-attributed deaths [14]. Life years lost were derived for mortalities observed in the study population using US Department of Health and Human Services Life Tables [15]. Each life year lost was adjusted with the appropriate age- and gender-specific utility value to determine the all-cause quality-adjusted life years (QALYs) lost by patient; these were summed to annual totals for each risk and age group [16]. The all-cause QALYs lost were then multiplied by the proportion of estimated influenza-attributed deaths from the rates found through the modeling technique described above to determine influenza-attributed QALYs lost. Lastly, the value of influenza-attributed QALYs was calculated by multiplying the annual estimate of QALYs lost for each risk and age group with the value of $150,000 per QALY [17].

### Removal of outliers

In order to further increase the accuracy of estimated medical costs attributable to influenza, and to reduce the impact that erroneous data and rare-but-extreme values might have on the estimates, we imposed restrictions to the dataset [18]. Outliers for length of stay and medical costs were excluded using statistical distributions equivalent to at least two standard deviations from the mean in a normal distribution. These calculated exclusion criteria are as follows: (1) ED visits which cost less than $100 (below 1^st^ percentile) or more than $1,500 (above 99^th^ percentile); (2) hospitalizations which cost less than $2,000 (below 2^nd^ percentile) or more than $95,000 (above 98^th^ percentile); (3) hospitalizations with a length of stay over 28 days (above 98^th^ percentile); and, (4) hospitalizations followed by extended care with a length of stay over 450 days (above 98^th^ percentile).

### Respiratory or circulatory causes of death

Influenza-attributed all-cause mortality estimates, as generated from the analysis described above, include those that may not be directly associated with influenza, such as accidental deaths. To provide more accurate estimates, deaths were categorized as those with a respiratory or circulatory cause and those without; deaths with a cardiorespiratory cause are presumed to be more directly associated with influenza. Because cause of death was not available for the majority of deaths, we instead applied an adjustment to the all-cause mortality model, which involved multiplying the observed deaths per week by the average proportion of deaths per calendar week due to respiratory or circulatory conditions. The average proportion was calculated from a random sample of 13,000 VA enrolled Veterans for whom cause of death data were available from 2010 to 2014. Furthermore, by calculating the proportion per calendar week, we were able to account for seasonal variation in the cardiorespiratory proportion of all mortality.

### Alternative values of QALY

Death associated with influenza is a major driver of the total annual economic burden on the US. Therefore, the impact of a range of valuations for the monetary value of a QALY on the total annual economic burden estimates were also explored. To account for varying opinions on the value of a QALY, four alternate values of $250,000, $200,000, $100,000 and $50,000 per QALY were applied to the economic burden of influenza-attributed mortality calculations [16,19].

## Results

Over the five-year observation period, the number of patients in the study population rose for each respiratory season from 5,294,641 patients in 2010 to 5,754,615 patients in 2014 –an increase of nearly 9% (S2 Table). The proportion of patients that identified as female increased slightly from 9.0% in 2010 to 9.7% in 2014. The proportion of patients aged 65 or older increased as well from 48.2% in 2010 to 53.7% in 2014 (p <0.0001). Notably, the proportion of patients classified as at high-risk for influenza-related complications averaged 11.0% for the 18 to 49 age group, 36.5% for the 50 to 64 cohort and 49.3% for the 65 and older group, decreasing slightly by 1%, 4%, and 1% for each, respectively, over the five years.

### Influenza-attributed outcomes

Annual mean influenza-attributed outcome estimates averaged over the five year study period were determined to be 10,674 (95% CI 8,661–12,687) for VA ED visits, 2,538 (95% CI 2,112–2,964) for VA hospitalizations, 5,522 (95% CI 4,834–6,210) for all-cause deaths and 3,793 (95% CI 3,375–4,211) for respiratory or circulatory deaths (Tables 1–5, S1 and S2 Figs). Overall, 4.4% of ED visits were attributed to patients aged 18 to 49, 30.0% by those aged 50 to 64, and 65.6% by those aged 65 and older. Patients aged 18 to 49, and 50 to 64, accounted for 5.0% and 14.9%, respectively, of the observed hospitalizations; the patients aged 65 and older were responsible for the remaining 80.1% of hospitalizations. Only 1.8% of influenza-attributed deaths befell patients aged 18 to 49, while 18.9% befell those aged 50 to 64 and 79.3% aged 65 and older. The proportions of influenza-attributed deaths from all-cause deaths were calculated to be 9.2%, 6.3% and 4.1% for those aged 18 to 49, 50 to 64, and 65 and older, respectively, in the high-risk group. In the low-risk group, the proportions were respectively 0.05%, 0.29% and 0.73% (Table 5).

**Table 1:** Annual estimates of influenza-attributed ED visits^1^ by risk and age group averaged over five respiratory seasons.

Risk	Age	N	95% CI		Person-periods	Rate per 100,000 person-periods	95% CI	
			Lower	Upper			Lower	Upper
High	18–49	186	132	241	128,037	146	103	188
	50–64	2,843	2,348	3,339	553,798	513	424	603
	65+	6,702	5,608	7,796	1,408,113	476	398	554
	*Total*	*9*,*732*	*8*,*087*	*11*,*376*	*2*,*089*,*948*	*466*	*387*	*544*
Low	18–49	291	180	401	1,040,587	28	17	39
	50–64	354	221	486	964,133	37	23	50
	65+	298	172	424	1,445,967	21	12	29
	*Total*	*942*	*574*	*1*,*311*	*3*,*450*,*687*	*27*	*17*	*38*
Both	18–49	477	312	642	1,168,625	41	27	55
	50–64	3,197	2,569	3,825	1,517,931	211	169	252
	65+	7,000	5,780	8,220	2,854,080	245	203	288
	*Total*	*10*,*674*	*8*,*661*	*12*,*687*	*5*,*540*,*636*	*193*	*156*	*229*

Due to rounding, numbers presented may not add up precisely to the totals provided and percentages may not precisely reflect the absolute figures.

^1^Events included here are those with a principal diagnosis of underlying respiratory or circulatory conditions (ICD-9-CM codes 390–519).

**Table 2:** Annual estimates of influenza-attributed hospitalizations^1^ by risk and age group averaged over five respiratory seasons.

Risk	Age	N	95% CI		Person-periods	Rate per 100,00 person-periods	95% CI	
			Lower	Upper			Lower	Upper
High	18–49	127	110	143	128,037	99	86	112
	50–64	374	301	446	553,798	67	54	81
	65+	2,029	1,706	2,351	1,408,113	144	121	167
	*Total*	*2*,*529*	*2*,*118*	*2*,*940*	*2*,*089*,*948*	*121*	*101*	*141*
Low	18–49	1	0	3	1,040,587	0	0	0
	50–64	3	0	10	964,133	0	0	1
	65+	4	0	12	1,445,967	0	0	1
	*Total*	*9*	*0*	*24*	*3*,*450*,*687*	*0*	*0*	*1*
Both	18–49	128	110	145	1,168,625	11	9	12
	50–64	377	299	456	1,517,931	25	20	30
	65+	2,033	1,702	2,363	2,854,080	71	60	83
	*Total*	*2*,*538*	*2*,*112*	*2*,*964*	*5*,*540*,*636*	*46*	*38*	*53*

Due to rounding, numbers presented may not add up precisely to the totals provided and percentages may not precisely reflect the absolute figures.

^1^Events included here are those with a principal diagnosis of underlying respiratory or circulatory conditions (ICD-9-CM codes 390–519).

**Table 3:** Annual estimates of influenza-attributed all-cause mortality by risk and age group averaged over five respiratory seasons.

Risk	Age	N	95% CI		Person-periods	Rate per 100,000 person-periods	95% CI	
			Lower	Upper			Lower	Upper
High	18–49	100	91	109	128,037	78	71	85
	50–64	1,017	898	1,137	553,798	184	162	205
	65+	3,940	3,485	4,394	1,408,113	280	247	312
	*Total*	*5*,*057*	*4*,*474*	*5*,*641*	*2*,*089*,*948*	*242*	*214*	*270*
Low	18–49	1	0	2	1,040,587	0	0	0
	50–64	23	17	29	964,133	2	2	3
	65+	441	343	538	1,445,967	30	24	37
	*Total*	*465*	*360*	*570*	*3*,*450*,*687*	*13*	*10*	*17*
Both	18–49	101	91	111	1,168,625	9	8	10
	50–64	1,041	915	1,166	1,517,931	69	60	77
	65+	4,380	3,828	4,933	2,854,080	153	134	173
	*Total*	*5*,*522*	*4*,*834*	*6*,*210*	*5*,*540*,*636*	*100*	*87*	*112*

Due to rounding, numbers presented may not add up precisely to the totals provided and percentages may not precisely reflect the absolute figures.

**Table 4:** Annual estimates of influenza-attributed mortality adjusted for respiratory or circulatory cause of death by risk and age group averaged over five respiratory seasons.

Risk	Age	N	95% CI		Person-periods	Rate per 100,000 person-periods	95% CI	
			Lower	Upper			Lower	Upper
High	18–49	38	34	42	128,037	30	27	33
	50–64	516	455	577	553,798	93	82	104
	65+	2,786	2,513	3,060	1,408,113	198	178	217
	*Total*	*3*,*341*	*3*,*003*	*3*,*679*	*2*,*089*,*948*	*160*	*144*	*176*
Low	18–49	10	8	12	1,040,587	1	1	1
	50–64	9	6	13	964,133	1	1	1
	65+	432	358	506	1,445,967	30	25	35
	*Total*	*452*	*372*	*532*	*3*,*450*,*687*	*13*	*11*	*15*
Both	18–49	49	43	54	1,168,625	4	4	5
	50–64	526	461	590	1,517,931	35	30	39
	65+	3,219	2,871	3,566	2,854,080	113	101	125
	*Total*	*3*,*793*	*3*,*375*	*4*,*211*	*5*,*540*,*636*	*68*	*61*	*76*

Due to rounding, numbers presented may not add up precisely to the totals provided and percentages may not precisely reflect the absolute figures.

**Table 5:** Annual estimates of influenza-attributed all-cause mortality and influenza-attributed mortality adjusted for respiratory or circulatory cause of death as the proportion of all deaths by risk and age group averaged over five respiratory seasons.

Risk	Age	N, Influenza Attributed Deaths		N, All Deaths	Proportion (%) of All	
		All-cause	Adjusted		All-cause	Adjusted
High	18–49	100	38	1,086	9.21	3.50
	50–64	1,017	516	16,062	6.33	3.21
	65+	3,940	2,786	97,028	4.06	2.87
	*Total*	*5*,*057*	*3*,*341*	*114*,*176*	4.43	2.93
Low	18–49	1	10	2,214	0.05	0.45
	50–64	23	9	7,986	0.29	0.11
	65+	441	432	60,068	0.73	0.72
	*Total*	*465*	*452*	*70*,*268*	0.66	0.64
Both	18–49	101	49	3,299	3.06	1.49
	50–64	1,041	526	24,048	4.33	2.19
	65+	4,380	3,219	157,096	2.79	2.05
	*Total*	*5*,*522*	*3*,*793*	*184*,*444*	2.99	2.06

Due to rounding, numbers presented may not add up precisely to the totals provided and percentages may not precisely reflect the absolute figures.

### Cost per influenza-attributed encounter

Annual mean estimates of direct medical costs for influenza-attributed ED visits averaged over the five year study period were similar across both risk and age groups, ranging from $592 in the high-risk 65 and older age group to $568 in the low-risk 50 to 64 age group (Table 6). For hospitalizations without extended care, the mean direct medical costs increased across age groups for both the high- and low-risk groups. In the high-risk group, the means were $13,673, $14,623 and $14,839 for those aged 18 to 49, 50 to 64 and 65 and older, respectively. In the low-risk group, the respective means were $10,872, $13,705 and $14,723. The mean direct medical costs varied across both risk and age groups for hospitalizations with extended care, ranging from $49,058 in the high-risk 18 to 49 age group to $73,233 in the low-risk 50 to 64 age group. The means were highest for the 50 to 64 age groups in both risk categories.

**Table 6:** Annual estimates of influenza-attributed direct medical costs for healthcare encounters by risk and age group in US Dollars averaged over five respiratory seasons.

Risk	Age	ED Visit			Hospitalization Only			Hospitalization with Extended Care		
		Mean	95% CI		Mean	95% CI		Mean	95% CI	
			Lower	Upper		Lower	Upper		Lower	Upper
High	18–49	574	570	577	13,673	12,959	14,386	49,058	18,164	79,951
	50–64	571	570	573	14,623	14,400	14,846	70,701	63,923	77,480
	65+	592	591	593	14,839	14,720	14,958	63,921	61,309	66,534
Low	18–49	569	567	571	10,872	10,301	11,442	61,688	37,830	85,545
	50–64	568	566	570	13,705	13,339	14,072	73,233	64,085	82,381
	65+	590	589	592	14,723	14,454	14,992	60,526	55,570	65,482

Ninety six percent of influenza-attributed hospitalizations resulted in a discharge to home or death, while the remaining 4% were followed by extended care at rehabilitation and/or skilled nursing facilities. The mean length of stay averaged over the five year study period for hospitalizations without extended care ranged from 4 to 6 days across the risk and age groups; for hospitalizations with extended care, the mean length of stay ranged from 31 days in the high-risk 18 to 49 age group to 50 days in the low-risk 50 to 64 age group (Table 7).

**Table 7:** Mean length of stay for hospitalizations without and with extended care^1^ by risk and age group in days averaged over five respiratory seasons.

Risk	Age	Hospitalization Only				Hospitalization with Extended Care			
		N	Mean	95% CI		N	Mean	95% CI	
				Lower	Upper			Lower	Upper
High	18–49	1,368	4.5	4.3	4.7	11	31	7	56
	50–64	14,724	4.9	4.9	5	393	47	41	53
	65+	47,811	5.2	5.2	5.3	1,984	41	39	44
Low	18–49	1,575	3.8	3.7	4	15	35	18	52
	50–64	5,370	4.8	4.7	4.9	110	50	41	59
	65+	10,114	5.4	5.3	5.5	453	43	38	48

^1^Events included here are those with a principal diagnosis of underlying influenza and pneumonia (ICD-9-CM codes 480–487).

### Economic burden of seasonal influenza epidemics in the VA population

Projected annual estimates of lost productivity for influenza-attributed ED visits, hospitalizations, hospitalizations with extended care and all-cause mortality averaged over the five year study period were $590 (95% CI $487–693) thousand, $626 (95% CI $517–746) thousand, $197 (95% CI $143–261) thousand and $25,758 (95% CI $22,600–28,909) thousand, respectively (Table 8). While those aged 65 and older carried the majority of the lost productivity burden caused by hospitalizations, hospitalizations with extended care and mortality (56%, 53% and 52%, respectively), those aged 50 to 64 carried the majority of the lost productivity burden caused by ED visits (57%).

**Table 8:** Annual estimates of lost productivity for influenza-attributed healthcare encounters and all-cause mortality by age group in thousands of US Dollars averaged over five respiratory seasons.

Age	ED Visit			Hospitalization Only			Hospitalization with Extended Care			All-cause Mortality		
	Mean	95% CI		Mean	95% CI		Mean	95% CI		Mean	95% CI	
		Lower	Upper		Lower	Upper		Lower	Upper		Lower	Upper
18–49	25.0	17.7	32.4	69.1	57.7	81.6	10.0	3.5	18.2	1,221	1,100	1,342
50–64	335	277	394	207	165	251	82	57	111	11,049	9,712	12,376
65+	229	192	267	350	293	413	105	83	132	13,488	11,788	15,191
*Total*	*590*	*487*	*693*	*626*	*517*	*746*	*197*	*143*	*261*	*25*,*758*	*22*,*600*	*28*,*909*

Due to rounding, numbers presented may not add up precisely to the totals provided and percentages may not precisely reflect the absolute figures.

Projected annual estimates of direct medical costs for influenza-attributed ED visits, hospitalizations, and hospitalizations with extended care averaged over the five year study period were $6.2 (95% CI $5.1–7.4) million, $36.1 (95% CI $29.8–42.7) million and $5.5 (95% CI $4.4–6.8) million, respectively (Table 9). Overall, 4.4% of ED visit costs were accounted for by those aged 18 to 49, 29.2% by those aged 50 to 64, and 66.4% by those aged 65 and older. For hospitalizations without extended care costs, patients aged 18 to 49 and those aged 50 to 64 accounted for 4.7% and 15.1%, and those aged 65 and older accounted for 80.2%. Finally, 2.3% of hospitalizations with extended care costs were accounted for by those aged 18 to 49, 4.0% by those aged 50 to 64, and 93.7% by those aged 65 and older.

**Table 9:** Annual estimates of influenza-attributed direct medical costs for healthcare encounters by risk and age group in thousands of US Dollars averaged over five respiratory seasons.

Risk	Age	ED Visit			Hospitalization Only			Hospitalization with Extended Care		
		Mean	95% CI		Mean	95% CI		Mean	95% CI	
			Lower	Upper		Lower	Upper		Lower	Upper
High	18–49	107	75	139	1,702	1,397	2,016	125	40	229
	50–64	1,623	1,338	1,913	5,425	4,300	6,568	211	153	276
	65+	3,968	3,314	4,623	28,909	24,112	33,765	5,168	4,167	6,232
	*Total*	*5*,*698*	*4*,*728*	*6*,*675*	*36*,*036*	*29*,*808*	*42*,*350*	*5*,*503*	*4*,*361*	*6*,*737*
Low	18–49	166	102	229	11	0	34	1	0	2
	50–64	201	125	277	39	0	135	9	0	35
	65+	176	101	251	57	0	175	6	0	20
	*Total*	*542*	*328*	*757*	*107*	*0*	*344*	*16*	*0*	*58*
Both	18–49	272	177	368	1,712	1,397	2,050	126	40	232
	50–64	1,824	1,463	2,190	5,465	4,300	6,703	220	153	311
	65+	4,143	3,416	4,874	28,966	24,112	33,940	5,174	4,167	6,253
	*Total*	*6*,*240*	*5*,*056*	*7*,*432*	*36*,*143*	*29*,*808*	*42*,*693*	*5*,*520*	*4*,*361*	*6*,*796*

Due to rounding, numbers presented may not add up precisely to the totals provided and percentages may not precisely reflect the absolute figures.

The annual monetary value of QALYs lost caused by influenza-attributed all-cause deaths averaged over the five year study period amounted to $1.1 (95% CI $1.0–1.2) billion (Table 10). The proportion of the monetized QALY loss across risk and age groups was highest for those aged 50 to 64 (71.5% for high-risk and 82.3% for low-risk), followed next by those aged 18 to 49 (27.9% for high and 15.6% for low risk), and smallest for those aged 65 and older (0.53% for high and 2.1% for low). The total annual economic burden, determined by adding the estimates of lost productivity, direct medical costs and the monetary value of QALYs lost, was $1.2 (95% CI $1.0–1.3) billion (Table 11).

**Table 10:** Annual estimates of influenza-attributed life years lost, quality adjusted life years (QALYs) lost and monetary value of QALYs lost by risk and age group in millions of US Dollars averaged over five respiratory seasons.

Risk	Age	Life Years Lost			QALYs Lost			Value of QALYs Lost		
		N	95% CI		N	95% CI		Mean	95% CI	
			Lower	Upper		Lower	Upper		Lower	Upper
High	18–49	2,686	2,444	2,927	2,009	1,829	2,190	301	274	329
	50–64	6,423	5,672	7,181	5,139	4,538	5,746	771	681	862
	65+	25.1	22.2	27.9	38.2	33.8	42.6	5.7	5.1	6.4
	*Total*	*9*,*134*	*8*,*138*	*10*,*136*	*7*,*187*	*6*,*401*	*7*,*979*	*1*,*078*	*960*	*1*,*197*
Low	18–49	27	0.0	53	25	0.0	50	3.8	0.0	7.5
	50–64	168	124	211	136	101	171	20	15	26
	65+	2.0	1.6	2.5	3.1	2.4	3.7	0.5	0.4	0.6
	*Total*	*196*	*126*	*267*	*164*	*103*	*226*	*25*	*15*	*34*
Both	18–49	2,712	2,444	2,981	2,035	1,829	2,241	305	274	336
	50–64	6,591	5,796	7,393	5,275	4,639	5,917	791	696	888
	65+	27.1	23.7	30.4	41.3	36.2	46.3	6.2	5.4	7.0
	*Total*	*9*,*330*	*8*,*263*	*10*,*404*	*7*,*351*	*6*,*503*	*8*,*204*	*1*,*103*	*976*	*1*,*231*

Due to rounding, numbers presented may not add up precisely to the totals provided and percentages may not precisely reflect the absolute figures.

**Table 11:** Annual estimates of economic burden using direct medical costs, lost productivity and monetary value of quality adjusted life years (QALYs) lost based on all-cause influenza-attributed mortality by age group in millions of US Dollars averaged over five respiratory seasons.

Age	Direct Medical Costs			Lost Productivity			Value of QALYs Lost			Total Economic Burden		
	Mean	95% CI		Mean	95% CI		Mean	95% CI		Mean	95% CI	
		Lower	Upper		Lower	Upper		Lower	Upper		Lower	Upper
18–49	2.1	1.6	2.6	1.3	1.2	1.5	305	274	336	309	277	340
50–64	7.5	5.9	9.2	12	10	13	791	696	888	810	712	910
65+	38	32	45	14	12	16	6.2	5.4	7.0	59	49	68
*Total*	*48*	*39*	*57*	*27*	*24*	*31*	*1*,*103*	*976*	*1*,*231*	*1*,*178*	*1*,*038*	*1*,*318*

Due to rounding, numbers presented may not add up precisely to the totals provided and percentages may not precisely reflect the absolute figures.

### Sensitivity analyses

Using a combination of the lowest valuation of a QALY and respiratory or circulatory causes of death, the total annual economic burden amounted to $251 (95% CI $217–285) million. Alternatively, when using all-cause mortality and the highest valuation of a QALY, the estimate totaled $1.9 (95% CI $1.7–2.1) billion (Table 12).

**Table 12:** Sensitivity analysis to estimate the annual economic burden using direct medical costs, lost productivity and monetary value of quality adjusted life years (QALYs) lost for different monetary values per QALY by all-cause and respiratory or circulatory cause of death adjusted influenza-attributed mortality and age group in millions of US Dollars.

Mortality	Age	$250,000 per QALY			$200,000 per QALY			$150,000 per QALY			$100,000 per QALY			$50,000 per QALY		
		Mean	95% CI		Mean	95% CI		Mean	95% CI		Mean	95% CI		Mean	95% CI	
			Lower	Upper		Lower	Upper		Lower	Upper		Lower	Upper		Lower	Upper
All-cause	18–49	512	460	564	410	369	452	309	277	340	207	186	228	105	94	116
	50–64	1,338	1,176	1,502	1,074	944	1,206	810	712	910	547	480	614	283	248	318
	65+	63	53	73	61	51	70	59	49	68	57	48	66	55	46	63
	*Total*	*1*,*913*	*1*,*689*	*2*,*139*	*1*,*545*	*1*,*364*	*1*,*728*	*1*,*178*	*1*,*038*	*1*,*318*	*810*	*713*	*908*	*443*	*388*	*498*
Adjusted	18–49	257	223	290	206	179	233	155	135	175	104	91	118	54	46	61
	50–64	679	595	764	546	478	615	413	362	465	280	245	315	147	128	166
	65+	56	48	65	55	46	63	53	45	62	52	44	60	50	42	59
	*Total*	*992*	*866*	*1*,*119*	*807*	*704*	*911*	*621*	*542*	*702*	*436*	*379*	*494*	*251*	*217*	*285*

## Discussion

Building on previously published research methods for estimating influenza-attributed urgent healthcare utilization (ED visits and hospitalizations), mortality and costs, this investigation presents an up-to-date assessment of the annual seasonal influenza epidemic burden in the US Veterans Affairs population. As the largest integrated healthcare system in the US, services provided to Veterans at VA can be followed across the care continuum from the non-urgent outpatient clinic to the ED and subsequent hospitalization, as well as post-discharge extended care in rehabilitation and nursing facilities. The analytical dataset used here therefore encompasses the majority of a patient’s VA healthcare and costs. We found 10,674 VA ED visits with costs amounting to $6.2 million, 2,538 VA hospitalizations with costs amounting to $41.7 million and 5,522 all-cause deaths with monetary losses amounting to $1.1 billion averaged over the five year study period from 2010 to 2014. Combined, these result in an estimated total annual influenza-attributed societal burden of $1.2 billion.

Consistent with previously reported results on the general US population, the annual burden of illness caused by seasonal influenza virus infections varied by both risk and age groups and was found to be greatest for those aged 65 and older and at high-risk for complications as based on the presence of certain co-existing medical conditions [1–7]. While ED visits and costs were closer among the three age groups than the other outcome event categories, overall, they differed greatly by risk, with those aged 65 and older at high-risk accounting for more of the visits than those aged 18 to 49 at low-risk. Similar findings have been reported by Schanzer and colleagues for the Canadian population [20]. The majority of the hospitalizations and deaths occurred for patients aged 65 and older, regardless of risk stratification; thus, most of the hospitalization costs and productivity losses were incurred by those aged 65 and older. However, the monetary value of QALYs lost was considerably smaller for this group than for both the 18 to 49 and 50 to 64 age groups. Overall, premature death was found to be the largest driver of costs, followed by hospitalization.

### Limitations

First, the negative-binomial model used in our analysis was thought to be most appropriate for the over-dispersed data under study and is otherwise similar to the Serfling-Poisson model that has been employed more widely for these types of estimations. Limitations of these methods have been described previously and include (1) the accuracy of identifying outcome events based on the presence of a principal diagnosis code from an observed healthcare encounter, which may not account for influenza’s contribution to prompt an individual to seek urgent or emergent care for other conditions or events that are therefore coded as the primary diagnosis; (2) the dependence on coding practices to identify observed outcome events, which may be influenced by a lack of coding resources (e.g. time and skill); and (3) the attribution of all excess outcomes to influenza when confounding, such as the presence of other respiratory viruses like the human metapneumovirus, is possible and may result in an over-estimation of the outcome events [2–7,20–21]. In addition, our estimation of underlying respiratory or circulatory deaths attributable to influenza, although based on a large random sample, is potentially susceptible to bias as we have not verified that the sample is representative of all VA deaths. Lastly, outliers in the cost data were excluded here in order to mitigate our concerns surrounding the impact that erroneous data and rare-but-extreme values may have on the estimates.

Second, it is difficult to compare the results found through this analysis directly with those reported in previously published studies. The US Veterans Affairs population differs from the US general population in terms not only of size (and growth over time), but in age, gender, and health status [8]. Furthermore, the healthcare systems differ in terms of organization and cost across populations under study. The estimates are reported as annual means over a five-year observation period from 2010 to 2014; many of the related earlier studies were performed with data through respiratory season 2010 only and not from the VA population [2–7].

Third, our methods for cost accounting were more conservative than some of the previous similar studies. Most notably, costs were limited to the day of an ED visit and the acute or extended care portion of a hospitalization and discharge. Others, such as Molinari and colleagues, have used a window of two weeks prior to and up to 30 days following discharge for these calculations, as well as direct medical costs associated with an outcome of death [5]. Additionally, VA cost data represent actual expense figures, not insurance claims, which have been shown to be lower than the cost paid by CMS for similar services [22–24]. Also, our healthcare encounter data are partial, including those that occurred at VA facilities but not those at non-VA facilities [25]. In contrast, our mortality data are complete. As such, it is likely that our results underestimate the influenza-attributed healthcare encounters that Veterans experience, while they over-estimate the number of influenza-attributed deaths. Finally, VA-specific influenza surveillance data, cause of death and VA-specific utility values for calculating QALYs lost were not available when these analyses were performed. Furthermore, the proportion of employed hospitalized Veterans may be lower than the average we used, leading to a possible over-estimation of the economic burden; however, we did not include wages lost by the Veterans’ caregivers, which may, conversely, lead to an under-estimation of the economic burden.

## Conclusion

Using five respiratory seasons of data, this study provided an assessment of the burden of influenza epidemics in the US Veterans Affairs population. Over the study period we estimated over 13,000 VA influenza-attributed healthcare encounters, more than 5,000 influenza-attributed deaths, $48 million in direct medical costs to VA and a projected cost to society of $1.2 billion occur each year. Future research efforts to evaluate the impact of immunization on this burden and identify opportunities by which to enhance prevention efforts for this population are warranted.

## Supporting Information

S1 Fig Observed and model-predicted influenza-attributed all-cause deaths per week. (TIF)

S2 Fig Observed and model-predicted influenza-attributed deaths adjusted for respiratory or circulatory cause of death per week. (TIF)

S1 Table Overview of estimate parameters. (PDF)

S2 Table Demographics and healthcare utilization for VA study population (respiratory seasons 2010 to 2014). (PDF)
