# Supplementary figures and images for: The Annual Burden of Seasonal Influenza in the US Veterans Affairs Population
Source: PLoS One. 2017 Jan 3;12(1):e0169344. doi: 10.1371/journal.pone.0169344 (PMC5207669; doi:10.1371/journal.pone.0169344)

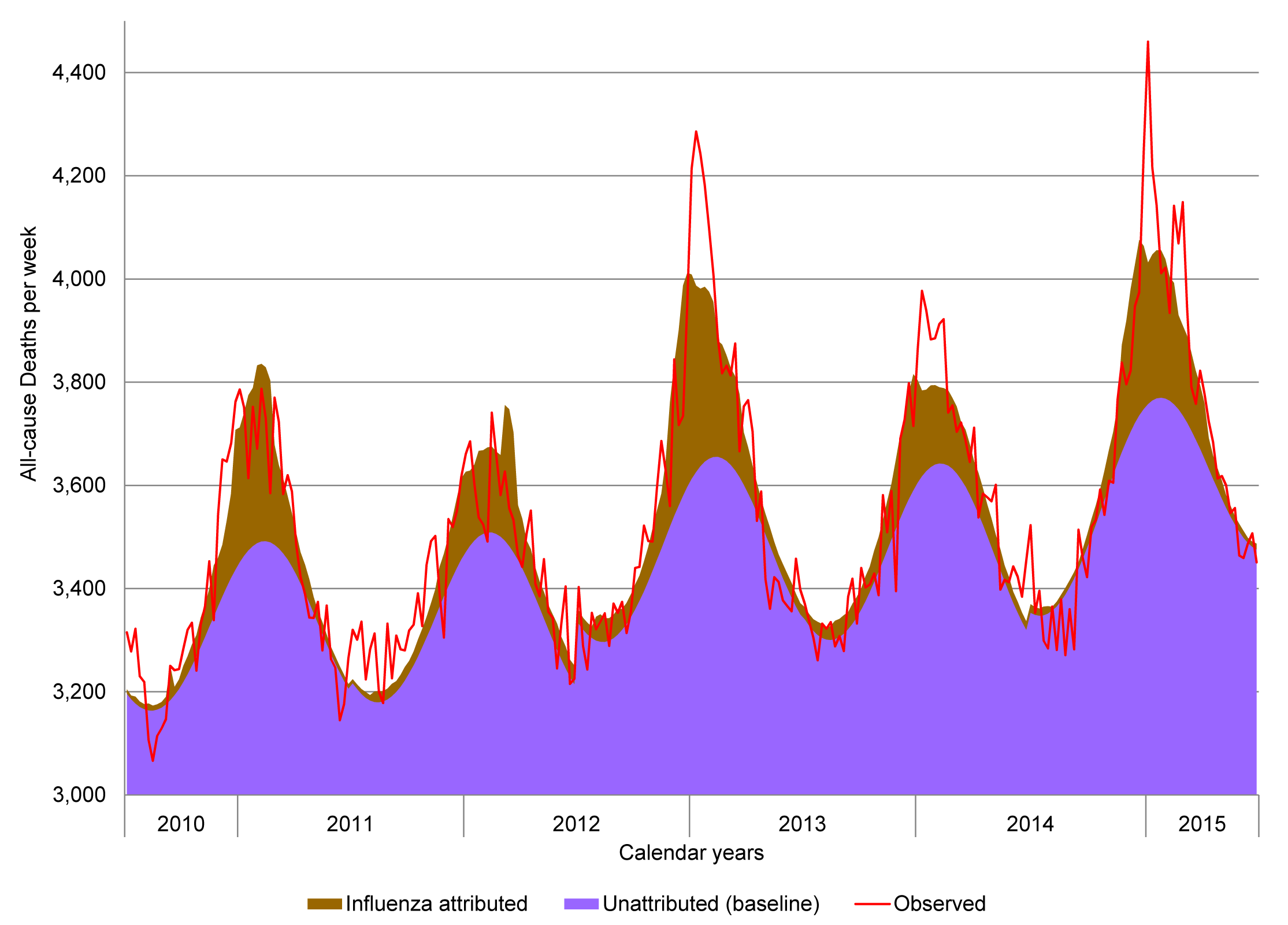

Supplement: S1 Fig — (TIF) [file pone.0169344.s001.tif]

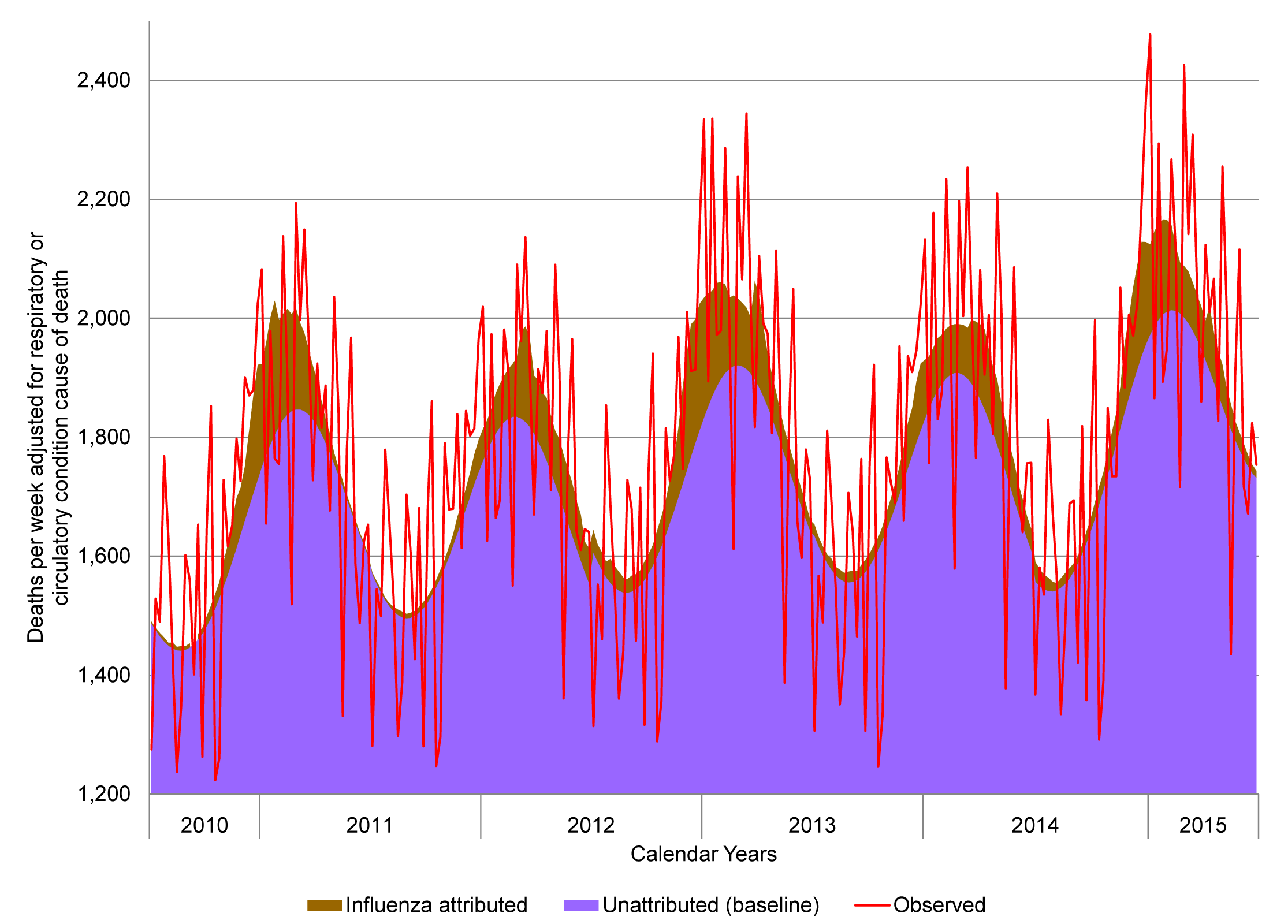

Supplement: S2 Fig — (TIF) [file pone.0169344.s002.tif]
