# Supplementary material for: The Annual Burden of Seasonal Influenza in the US Veterans Affairs Population
Source: PLoS One. 2017 Jan 3;12(1):e0169344. doi: 10.1371/journal.pone.0169344 (PMC5207669; doi:10.1371/journal.pone.0169344)
Supplement: S1 Table — (PDF) [file pone.0169344.s003.pdf]

Parameter				Source
High-risk for influenza-related complications	Chronic cardiac disease	Acute rheumatic fever	391–392	[4] Mullooly, et. al
		Chronic rheumatic heart disease	393–398	
		Hypertensive heart disease	402, 404	
		Ischemic heart disease	410–414	
		Diseases of pulmonary circulation	416, 417	
		Other forms of heart disease	421, 423, 424, 425, 427.1–427.5, 427.8, 428, 429	
		Atherosclerosis, polyarteritis nodosa	440, 446	
		Congenital anomalies	745–747	
		Surgical/device conditions	V42.1, V45.0, V45.81, V45.82	
		Cardiovascular syphilis	093	
		Candidal endocarditis	11281	
		Myocarditis due to toxoplasmosis	1303	
	Chronic pulmonary	Other metabolic and immunity disorders	277.0, 277.6	
		COPD and allied conditions	491–496	
		Pneumoconioses/other lung diseases due to external agents	500–506, 507.0, 507.1, 508	
		Other diseases of respiratory system	510, 513–517, 518.0–518.3, 519.0, 519.9	
		Congenital anomalies	748.4–748.6, 759.3	
		Lung transplant	V42.6	
		Tuberculosis	011, 012	
		Diseases due to other mycobacteria	031.0	
		Sarcoidosis	135	
	Chronic renal disease	Hypertensive renal disease	403	
		Nephritis, nephrotic syndrome, nephrosis	581–583, 585–587, 588.0, 588.1	
		Chronic pyelonephritis	590	
		Other specified disorders of kidney and ureter	593.8	
		Dialysis and transplant	V42.0, V45.1, V56	
	Diabetes mellitus	Diabetes mellitus	250, 251, 648.0	
		Complications of diabetes	357.2, 362.0, 362.11, 366.41	
	Hemoglobinopathies	Anemias	282–284	
	Immunosuppressive disorders	HIV/retroviral disease	042–044, 079.5, V08	
		Disorders involving immune mechanism	279	
		Diseases of blood and blood-forming organs	288.0, 288.1, 288.2	
		Polyarteritis nodosa	446	
		Diseases of musculoskeletal system and connective tissue	710.0, 710.2, 710.4, 714	
		Organ/tissue transplants	V42.0–V42.2, V42.6–V42.9	
		Radiation/chemotherapy	V58.0, V58.1	
	Malignancies		140–208	
	Other metabolic and immunity disorders	Disorders of adrenal glands	255,	
		Other disorders	270, 271, 277.2, 277.3, 277.5, 277.8	
	Liver diseases	Chronic liver disease and cirrhosis	571	
		Liver abscess and sequelae of chronic liver disease	572.1–572.8	

Parameter					Source
High-risk for influenza-related complications *(continued)*	Neurological / musculoskeletal	Psychotic conditions		290, 294.1	[5] Mullooly, et. al
		Mental retardation		318.1, 318.2	
		Hereditary and degenerative diseases of CNS		330, 331, 333.0, 333.4-333.9, 334, 335	
		Other disorders of CNS		340, 341, 343, 344.0	
		Disorders of peripheral nervous system		358.0, 358.1, 359.1, 359.2	
		Late effects of CVD		438	
		Chondrodystrophy		756.4	
Proportion of employed Veterans, 2014			18-49	73.8%	[11] US Bureau of Labor Statistics
			50-64	64.8%	
			65+	18.8%	
Mean daily occupational wage in US dollars, 2014			All	$182	[12] US Bureau of Labor Statistics
Utility scores		Male	20–29	0.922	[15] Hanmer, et. al
			30–39	0.912	
			40–49	0.880	
			50–59	0.853	
			60–69	0.827	
			70–79	0.788	
			80–89	0.757	
		Female	20–29	0.905	
			30–39	0.886	
			40–49	0.855	
			50–59	0.829	
			60–69	0.800	
			70–79	0.758	
			80–89	0.701	
Proportion of estimated influenza-attributed all-cause deaths in the mean annual number of all-cause deaths		High-risk	18-49	9.21%	This study
			50-64	6.33%	
			65+	4.06%	
		Low-risk	18-49	0.05%	
			50-64	0.29%	
			65+	0.73%	
Proportion of estimated influenza-attributed cardiovascular and respiratory (adjusted) deaths in the mean annual number of all-cause deaths		High-risk	18-49	3.50%	This study
			50-64	3.21%	
			65+	2.87%	
		Low-risk	18-49	0.45%	
			50-64	0.11%	
			65+	0.72%	
