# Supplementary material for: The Annual Burden of Seasonal Influenza in the US Veterans Affairs Population
Source: PLoS One. 2017 Jan 3;12(1):e0169344. doi: 10.1371/journal.pone.0169344 (PMC5207669; doi:10.1371/journal.pone.0169344)
Supplement: S2 Table — (PDF) [file pone.0169344.s004.pdf]

**Characteristic**			**2010**		**2011**		**2012**		**2013**		**2014**	
			N	%	N	%	N	%	N	%	N	%
Total patients			5,294,641		5,444,514		5,559,011		5,650,398		5,754,615	
Age	18-49		1,064,576	20.1%	1,112,468	20.4%	1,163,619	20.9%	1,222,516	21.6%	1,279,944	22.2%
	50-64		1,676,091	31.7%	1,600,396	29.4%	1,507,383	27.1%	1,423,041	25.2%	1,382,743	24.0%
	65+		2,553,974	48.2%	2,731,650	50.2%	2,888,009	52.0%	3,004,841	53.2%	3,091,928	53.7%
Gender	Female		476,099	9.0%	491,273	9.0%	512,994	9.2%	541,115	9.6%	559,929	9.7%
	Male		4,818,538	91.0%	4,953,231	91.0%	5,046,014	90.8%	5,109,278	90.4%	5,194,680	90.3%
	Other/Unknown		4	0.0%	10	0.0%	3	0.0%	5	0.0%	6	0.0%
Race	White		3,449,664	65.2%	3,569,795	65.6%	3,653,524	65.7%	3,704,069	65.6%	3,766,459	65.5%
	African-American		742,366	14.0%	786,905	14.5%	823,838	14.8%	856,132	15.2%	889,132	15.5%
	Hispanic		258,319	4.9%	272,393	5.0%	285,753	5.1%	299,646	5.3%	316,504	5.5%
	Other/Missing		844,292	15.9%	815,421	15.0%	795,896	14.3%	790,551	14.0%	782,520	13.6%
Region*	West		863,876	16.3%	889,696	16.3%	914,541	16.5%	938,857	16.6%	971,830	16.9%
	Central		1,322,067	25.0%	1,360,006	25.0%	1,394,885	25.1%	1,412,265	25.0%	1,432,860	24.9%
	South		1,868,141	35.3%	1,940,617	35.6%	1,988,212	35.8%	2,027,728	35.9%	2,073,877	36.0%
	East		1,240,557	23.4%	1,254,195	23.0%	1,261,373	22.7%	1,271,548	22.5%	1,276,048	22.2%
Utilization	Patients with at least one hospitalization		371,477	7.0%	375,596	6.9%	375,290	6.8%	375,782	6.7%	384,918	6.7%
	Total hospitalizations		573,935		580,243		573,839		575,649		591,935	
	Patients with at least one outpatient visit		4,657,684	88.0%	4,794,046	88.1%	4,908,231	88.3%	4,979,975	88.1%	5,085,233	88.4%
	Total outpatient visits		59,395,869		62,821,913		65,632,501		68,039,765		72,269,402	
	Median no. of outpatient visits per patient		7	3-15	7	3-16	7	3-16	7	3-17	8	3-18
Risk	High	18-49	123,537	2.3%	123,963	2.3%	126,979	2.3%	129,062	2.3%	136,645	2.4%
		50-64	640,472	12.1%	598,326	11.0%	550,984	9.9%	501,359	8.9%	477,849	8.3%
		65+	1,274,930	24.1%	1,351,893	24.8%	1,425,998	25.7%	1,472,111	26.1%	1,515,634	26.3%
	Low	18-49	941,039	17.8%	988,505	18.2%	1,036,640	18.6%	1,093,454	19.4%	1,143,299	19.9%
		50-64	1,035,619	19.6%	1,002,070	18.4%	956,399	17.2%	921,682	16.3%	904,894	15.7%
		65+	1,279,044	24.2%	1,379,757	25.3%	1,462,011	26.3%	1,532,730	27.1%	1,576,294	27.4%

*West: AK, AZ, CA, CO, HI, ID, MT, NM, NV, OR, UT, WA, WY; Central: AR, IA, IL, KS, LA, MN, MO, ND, NE, OK, SD, TX, WI
South: AL, Washington DC, FL, GA, KY, MS, NC, PR, SC, TN, VA, WV; East: CT, DE, IN, MA, MD, ME, MI, NH, NJ, NY, OH, PA, RI, VT
